# Trade-off between pollinator-wildflower diversity & grassland yields

**DOI:** 10.1038/s44185-024-00070-6

**Published:** 2025-01-20

**Authors:** Nicholas J. Balfour, Ciaran Harris, Jonathan Storkey, Francis L. W. Ratnieks

**Affiliations:** 1 Laboratory of Apiculture & Social Insects, Department of Ecology & Evolution, School of Life Sciences, University of Sussex, Brighton, UK; 2 Protecting Crops and the Environment, Rothamsted Research, Harpenden, Hertfordshire, UK

**Keywords:** Ecology, Agroecology

## Abstract

This is a critical moment for land use policy globally, with many countries (e.g. the UK and the European Union) currently undertaking significant green reforms of their agricultural policies. Despite their importance for maintaining agricultural outputs and plant diversity, the effects of artificial soil enrichment on pollinators remain poorly understood. Our two-year study at the world’s longest-running ecological experiment, Park Grass, Rothamsted, examines the relationship between soil fertilisation, grassland yield and biodiversity. Our data show a large and significant negative effect of the major plant nutrients (NPK) on the abundance, species richness and functional diversity of both pollinators and flowering plants. The results also indicate a large and significant trade-off between productivity and biodiversity. Our findings are a salutary reminder of the challenge in reconciling conflicting aims in farmland management and strongly suggest that financial incentives are necessary to offset yield reductions to improve biodiversity outcomes in agricultural grasslands.

## Introduction

Almost a quarter of the Earth’s land area is agricultural grassland, contributing to the livelihoods of over 800 million people^1^. Grassland fertilisation has boosted global food production^2^ but has come at the cost of environmental degradation and adverse effects on human health and welfare^3,4^. We now fix more reactive nitrogen every year, via the Haber–Bosch process, than all natural processes on land combined^5^. This has an enormous carbon footprint, accounting for 1.4% of global CO_2_ emissions^6^. Increasing soil fertility induces physical, chemical and biological changes often leading to bottom-up effects in local ecosystems. Thus soil eutrophication can lead to air pollution, marine and freshwater eutrophication and biodiversity loss, as well as favouring some invasive species. However, agricultural grasslands can potentially deliver ecosystem services, including pollination, natural pest control, greenhouse gas sequestration, water purification, as well as cultural value.

Agricultural grassland is the leading land use category in the UK (46%)^7^, far exceeding arable land (25%), urbanized areas (~10%), urban gardens, (~2%), or wooded areas (~10%)^8^. However, only 1–2% of UK grasslands are now considered high-quality species rich habitats^9^. This is due to the increased management intensity, especially from the middle of the 20th century, including soil fertilisation and seeding with yield-enhancing species, such as ryegrass.

Pollinators are vital to agricultural productivity and maintaining natural ecosystems^10,11^. However, declines in the abundance and distribution of many pollinator species have prompted concerns for their pollination services^12^. While several causes have been identified, agricultural intensification is considered a key factor^12–14^. Despite this, the effects of artificial soil enrichment on pollinators remain poorly understood^15^.

Increasing soil fertility is thought to affect pollinator communities via three principal mechanisms: (i) food plant quantity (through changes to community composition), (ii) nutritional quality of nectar and pollen, and (iii) flowering phenology^15^. However, there remains many knowledge gaps in these areas^16^. For example, soil enrichment is well known to impact plant competition dynamics, favouring fast-growing competitive grasses at the expense of flowering forbs and legumes^17,18^. This in turn may reduce flowering plant functional diversity^17^, which is known to negatively impact pollinator diversity^19,20^. However, to our knowledge, the direct link between soil enrichment and pollinator diversity is yet to be quantified.

The Park Grass Experiment at Rothamsted, southeast England, was set up in 1856 and is the world’s longest-running ecological experiment^2^. The original aim was to investigate ways to improve pasture productivity via organic and inorganic soil fertilisation. Subsequently, Park Grass has proved of great value in addressing a wide range of ecological, environmental and evolutionary questions^2,21^, and continues to be a valuable resource for gaining new insights into agroecological systems^22,23^. Data from Park Grass are particularly valuable as the fertiliser treatments have acted as filters on the original meadow’s plant community, meaning that the species now found on each plot are naturally assembled. However, the relationships between the changes in plant communities and the fauna that use them as a resource remain relatively under-studied. Here, in the first study of its kind at this site, we quantify the impact of soil fertilisation on pollinators and flowering plants at Park Grass.

## Results

### Pollinator Census

**Fig. 1:**
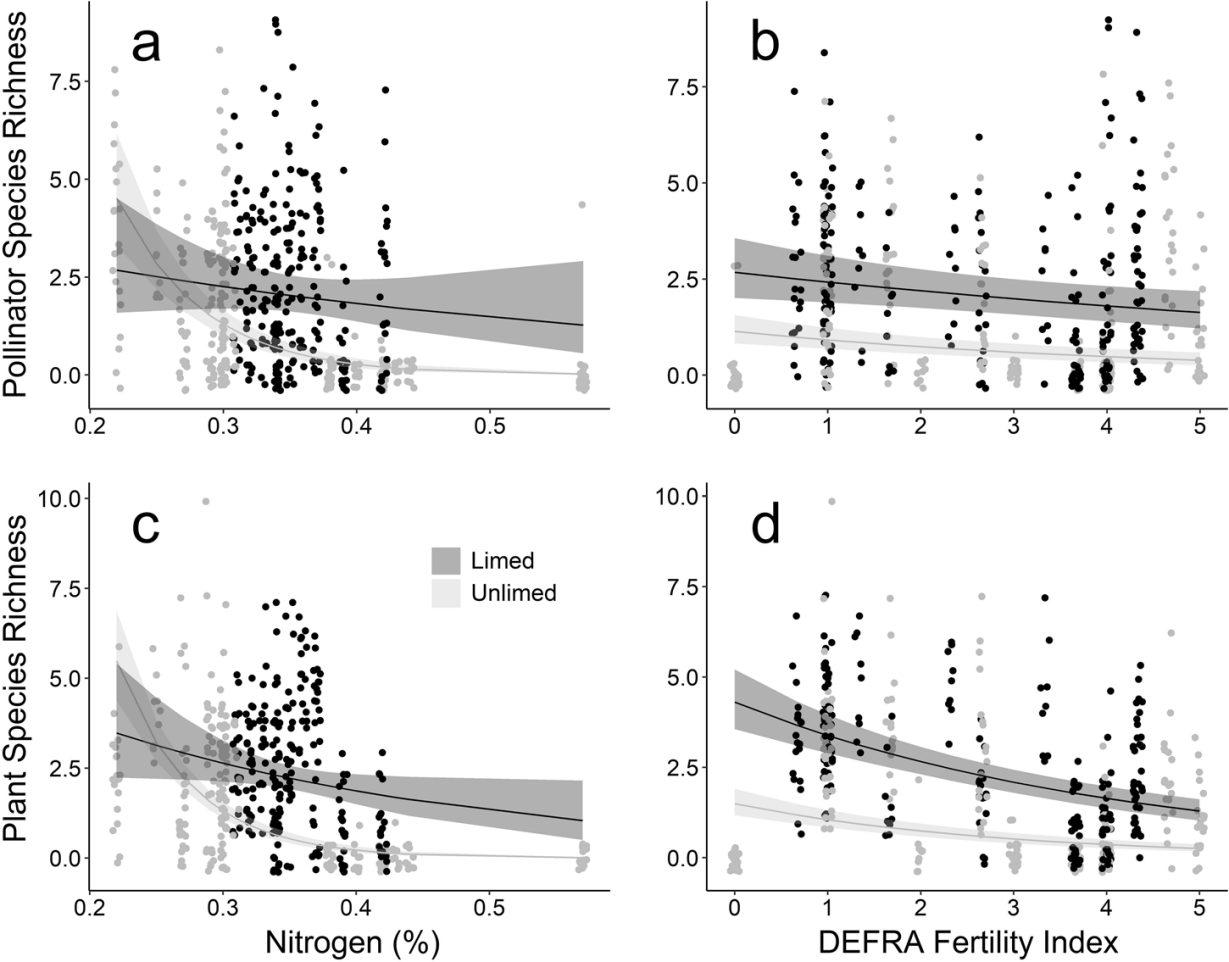
Negative impact of increasing soil fertility on flower and pollinator abundance and species richness. Model predictions of the relationship between pollinator species richness and soil nitrogen (%) (**a**) and mean DEFRA Fertility Index (**b**), and for flowering plant species richness and soil nitrogen (%) (**c**) and Mean DEFRA Fertility Index (**d**). Also shown are 95% Confidence Intervals (shaded areas) and raw data points per plot per study day (circles). Data are from plots receiving agricultural lime (dark grey) and those receiving no lime (light grey).

Across 1416 40 m^2^ transects surveys of flower-vising insects we recorded 1285 foraging individuals (Fig. 1a and b). In order of abundance these were bees 65.8% (honeybees 32.2%, bumble bees 29.0%, other bees 4.6%), flies 27.5% (6.5% hoverflies), beetles 2.9%, butterflies 2.1%, wasps 1.6%. Pollinator abundance and species richness were significantly negatively associated with the Defra Fertility Index (which is based on P, K and Mg; *P* < 0.001), N (*P* < 0.001) and lime application (*P* < 0.001), the interaction between lime application and the Defra Fertility Index and the interaction between lime application and N (nitrogen; Fig. 1, Table S2, Supplementary Information).

Honey bee (*Apis mellifera*) abundance was negatively associated with the Defra Fertility Index (*P* < 0.001), and N (*P* < 0.001), lime application (*P* = 0.003), and the interaction between the Defra Fertility Index and N (*P* < 0.001), and positively associated with the interaction between lime application and N (*P* < 0.001), and the interaction between lime application and the Defra Fertility Index (*P* = 0.047). Whereas non-honey bee abundance was significantly negatively associated with N (*P* < 0.001), and lime application (*P* < 0.001), and positively associated with the interaction between lime application and N (*P* < 0.001), but not with the Defra Fertility Index (*P* = 0.171).

### Flowering Plant Censuses

We recorded 7016 flower units (Fig. 1c and d, Table S1, Supplementary Information) in 2508 quadrats of 1 m^2^ from 19 flowering plant species. The most common were, *Pimpinella saxifraga* (21.1%), *L. corniculatus* (17.8%), *C. nigra* (16.0%), *Leontodon hispidus* (11.2%), *Trifolium pratense* (10.3%), *Scorzoneroides autumnalis* (8.1%), *Ranunculus auricomus* (4.0%), *Plantago lanceolata* (3.0%), *Achillea millefolium* (2.0%), *Knautia arvensis* (1.6%), *Betonica officinalis* (1.3%), *Cirsium vulgare* (1.2%), *Heracleum sphondylium* (1%), and six others (*Agrimonia eupatoria*, *Melilotus officinalis, G. verum, Lathyrus pratensis*, *T. repens*, and *Stellaria graminea*) in small numbers (<1%). Flowering plant abundance and species richness were significantly negatively associated with the Defra Fertility Index (*P* < 0.001), and N (*P* < 0.001), and lime addition (*P* < 0.001), and positively with the interaction between lime application and the Defra Fertility Index, and the interaction between lime application and N (Fig. 1, Table S2, Supplementary Information).

### Relationship Between Pollinators and Flowering Plants

*C. nigra* was the most commonly visited plant species (41.9% of pollinator visits), followed by *H. sphondylum* (14.7%), *K. arvensis* (7.7%), *L. hispidus* (7.0%), S. *autumnalis* (5.7%), *P. saxifraga* (5.0%), *T. pratense* (4.6%), *R. auricomus* (4.4%), *L. corniculatus* (4.0%), *A. millefolium* (4.0%), and *C. arvense* (1.9%), *B. officinalis* (<1%), *T. repens* (white clover; <1%).

Flowering plant abundance per plot was significantly positively related to both pollinator abundance (*R*^2^ = 0.774, t = 10.12, *P* < 0.001) and species richness (*R*^2^ = 0.59, t = 6.51, *P* < 0.001) per plot (Figure S1a). This was also the case for flowering plant species richness per plot and both pollinator abundance (*R*^2^ = 0.49, t = 5.53, *P* < 0.001) and species richness (*R*^2^ = 0.67, t = 7.93, *P* < 0.001) per plot (Figure S1b).

### Functional Diversity

Both flower (*P* < 0.001) and pollinator (*P* = 0.005) functional richness were significantly negatively associated with N (Table S2, Supplementary Information). Both flower (*P* < 0.001) and pollinator (*P* = 0.002) functional richness were also significantly positively associated with lime. However, only pollinator functional richness was significantly negatively associated with the DEFRA Fertility Index (*P* = 0.005).

### Cost-Benefit Analysis

Both mean flower (*R*^2^ = 0.11, *z* = −1.96, *P* = 0.049) and mean pollinator (*R*^2^ = 0.11, *z* = −2.55, *P* = 0.011) species richness were negatively associated with the DEFRA Fertility Index per plot (Fig. 3). By contrast, mean hay yield per plot was positively associated with this index (*R*^2^ = 0.26, *z* = 3.97, *P* < 0.001).

## Discussion

Our results show that fertiliser application reduced the abundance and species richness of flowering plants across the Park Grass experiment. This was the case with both of our indicators of fertility, the Defra Fertility Index (PKMg) and N (nitrogen; Fig. 1), and is due, primarily, to fertiliser application creating conditions that favour a limited number of fast-growing species, such as grasses^24^. Both flower abundance (5.18 times) and species richness (8.46 times) were severalfold greater in the two untreated plots than in the two receiving the greatest amount of fertiliser. Flower assemblages were directly impacted by fertilisation (Figure S2, Supplementary Materials). Plots receiving the highest levels of nitrogen had higher proportions of Apiaceae (e.g. *P. saxifraga*). Those receiving all nutrients except nitrogen (PKNaMg) were dominated by legumes (e.g. *T. pratense* and *L. corniculatus*) and had higher flower abundance than expected for the high fertility.

**Fig. 2:**
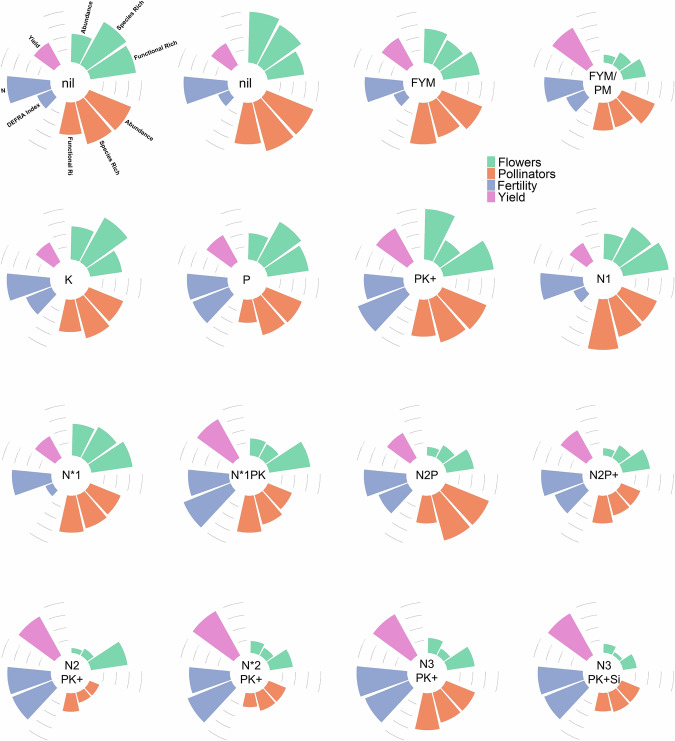
Relationship between pollinators, flowers, soil fertility and yield across fertiliser treatments. Radar diagrams showing, per Park Grass treatment plot, mean: flower abundance, flower species richness, flower functional diversity, pollinator abundance, pollinator species richness, pollinator functional richness, DEFRA Fertility Index, soil nitrogen, and hay yield. Treatments are given in the middle of each radar diagram. Each category has been scaled against the maximum value recorded across the plots (i.e. x’ = x/max(x)). Lines represent 0.25, 0.5, 0.75, and 1. Treatments: Nil plots: zero inputs, N*1, N*2: sodium nitrate supplying 48, 96 kg N/ha and 78, 157 kg Na/ha; N1, N2, N3: ammonium sulphate supplying 48, 96, 144 kg N/ha and 55, 110, 165 kg S/ha. Plus signs (+) indicate the addition of Na (sodium sulphate at 15 kg/ha Na and 10 kg/ha S) and Mg (magnesium sulphate at 10 kg/ha Mg and 13 kg/ha S). Si denotes water soluble sodium silicate supplying 135 kg/ha Si and 63 kg/ha Na. Organic fertiliser plots received 35 t/ha farmyard manure (FYM) and pelleted poultry manure (PM) every fourth year.

Strikingly, we found a near doubling in pollinator abundance (95% greater pollinator abundance) and richness (84% greater pollinator species richness) in the untreated plots versus those receiving high levels of fertilisers. Importantly, bees, which are key pollinators, were an order of magnitude more numerous (9.35 times) in the untreated plots than those receiving the highest levels of fertilisation. The plot receiving all nutrients except nitrogen (PKNaMg) was the exception to this trend, with a relatively high pollinator abundance and species richness (Fig. 2). All pollinator groups, including bees, were present on untreated plots and those receiving the lowest level of fertilisation. By contrast, plots receiving high levels of fertilisers were dominated by flies, Diptera, and beetles, Coleoptera. Despite previous work indicating that *A. mellifera* reacts differently to N inputs than other pollinator species^25^, we found that both groups were negatively impacted by high levels of fertiliser application (NPK).

Furthermore, pollinator abundance and species richness were positively related to both flower abundance and species richness per plot (Figure S1a, Supplementary Information). This illustrates the dependence of pollinators on flowering plants. These findings are in line with several previous studies that have shown that nutrient enrichment causes a loss of plant diversity^26,27^. In particular, legumes and other pollinator-visited plants are thought to be adversely affected by increased soil fertility^17,20,28^. Enhanced nitrogen levels in soils can also affect pollinators via their influence on floral traits. However, this area remains poorly understood^16^. Several studies have shown that low-level nitrogen application can increase plant flower production^29,30^. A similar pattern has also been observed with soil nitrogen and the quantity, and certain aspects of quality (e.g. amino acid content), of plant nectar and/or pollen production^31,32^. However, this effect appears to vary markedly between species^7^. Changes in floral phenology have also been observed with nitrogen addition, leading to potential phenological asynchrony with their associated pollinators^33,34^.

Interestingly, both flower and pollinator functional richness (the variety of traits within a community) were negatively correlated with nitrogen application (Table S2, Supplementary Information). Functional richness was greater for both flowers (100%) and pollinators (30%) in the untreated plots than those receiving the greatest level of fertilisation. This effect on plant functional richness has been observed previously in European grasslands^17,35^. Plant assemblages with distinct floral traits are considered necessary to support diverse pollinator communities^26^. It is generally assumed that this is because greater flower trait diversity facilitates more pollinator species to coexist via niche partitioning^36,37^. Greater pollinator functional diversity has also been shown to enhance crop pollination and yield^38^.

Over the last century nutrient enrichment from multiple sources has become an increasing threat to biodiversity, and the structure and stability of natural ecosystems^3^. Studies have shown, for example, that high concentrations of nutrients via soil fertilisation impact soil microbiota^39^ and cause reduced plant diversity and community structure^24,27^. However, the effect on pollinators has received relatively little research focus^16^, despite evidence suggesting a strong link between British bee and aculeate wasp extinctions and the introduction of first guano and then artificial fertilisers^13^.

The use of artificial fertilisers continues to increase globally and this expansion is now largely driven by agriculture across Asia^1^. A concomitant escalation in emissions of nitrogenous compounds from agricultural and industrial activities has also increased atmospheric nitrogen deposition in global natural and semi-natural ecosystems^40^. In recent decades fertiliser costs across the world have risen dramatically, primarily due to increasing energy costs in N fixation^41^. Consequently, this has increased economic uncertainty for farmers, and food prices for consumers.

Our analysis also indicates that increasing soil pH by applying calcium carbonate (agricultural lime), can mitigate, to a degree, the negative impact of artificial nitrogen application on flowering plant and pollinator populations (Fig. 1). Soil acidification affects plant communities by causing edaphic stress, which increases metal toxicity, cation leaching, and lowers soil nutrient availability^42^. In our study, limed plots had greater pollinator abundance (50%), pollinator species richness (70%), flower species richness (68%) and flowering species abundance (15%) than those not treated with lime.

**Fig. 3:**
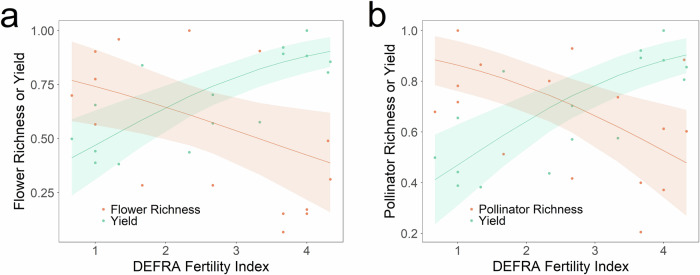
Trade-off between flower and pollinator species richness and grassland yield. Relationship between flower (**a**) or pollinator (**b**) species richness (orange circles), hay yield (green circles) and the DEFRA Fertility Index. Also shown are beta regressions (lines) and 95% confidence intervals (shaded areas). To determine the level of grasslands soil fertility that maximises both productivity and flowering plant and pollinator species richness we combined Park Grass hay yield and soil nutrient data with our plant and pollinator species richness data. All data have been normalised using the following formula: zi = (xi – min(x)) / (max(x) – min(x)).

Our most important and challenging finding is the existence of a trade-off between flower and pollinator diversity and grassland yield (Fig. 3 and Figure S4, Supplementary Materials). This shows that to maximise flower species richness, and hence also pollinator species richness, a significant drop in fertility is required and will reduce yield. Thus indicating that the subsidies proposed in the forthcoming UK and EU agricultural reforms are an economic necessity for land stewardship that reduces fertiliser application and its negative consequences for biodiversity. More generally, the existence of this trade-off means that it is necessary to consider both the costs and benefits involved, with the most important cost being reduced agricultural yield. Importantly, our data show the potential contribution of legumes, such as clover, which fix atmospheric N via their mutualism with *Rhizobium* bacteria that are housed and supplied with energy in their root nodules, in mitigating this trade-off.

Lastly, our data suggest that legume abundance was positively associated with soil P, K and Mg levels (i.e. the DEFRA Fertility Index), but negatively with soil N (Table S2, Supplementary Information). Plots fertilised with all nutrients except nitrogen (PKNaMg) selected for high proportions of leguminous species that maintained yields but also supported high numbers of pollinators, including bees. Although flower species richness was not as high as the plots with no fertiliser additions, legume-rich pastures represent an attractive solutionwhere managing grasslands for pollinator function is the primary aim, rather than maximising species richness per se^43,44^.

The multiplicity of environmental and socio-political demands on land use necessitates a rapid change to the management of landscapes and the multiple benefits they can potentially provide^45^. To this end, the UK and the European Union are currently undertaking significant green reforms of their agricultural policies. Data from naturally assembled plant communities of the type analysed here at the Park Grass Experiment are particularly valuable in this context as they provide evidence for management options that result in the sustainable co-existence of beneficial species as opposed to sowing ‘artificial’ species mixtures that may not persist, are often of non-native seed stock, and costly^46^.

Our results show significant biodiversity and pollination service benefits from reducing fertiliser inputs in agricultural grasslands. Reducing grassland production intensity has the potential to realise many of the aspirations of multifunctional landscape: by benefiting a wide range of taxa^47^ including pollinators, increasing resilience to extreme weather events, and ecosystem service delivery, such as increased natural pest control, soil health, air quality, and reduced soil erosion^48^. Importantly, it would also reduce the CO_2_ emissions resulting from the Haber–Bosch process, which is typically powered by natural gas. To realize these benefits, well-designed policies are needed to incentivize the sustainable management of pastoral landscapes. Our data indicate that soil nutrients management strategies that favour nitrogen-fixing legumes, i.e. low to zero N and intermediate P, K and Mg inputs, with lime addition can lessen the trade-off between biodiversity and yield in agricultural grasslands.

## Methods

### Study Site

The basic design and fertiliser treatments at Park Grass, Rothamsted, United Kingdom (51.804, -0.373) were set up over 150 years ago, although these have been somewhat modified. The design does not include the replication of plots which would certainly have been incorporated if it had been set up more recently. Nevertheless, the plots include two controls (i.e. zero inputs), four levels of nitrogen, from 48 to 144 kg per ha, applied as ammonium sulphate, (NH_4_)_2_SO_4_, or sodium nitrate, NaNO_3_, with various combinations of macro- and micro-nutrients (P, K, Na, Mg, Si). In addition, there are several organic fertiliser treatments. Within each treatment the four plots receive different levels of calcium carbonate (‘agricultural lime’) to counter the pH-lowering effect of ammonium sulphate application (Figure S3, Supplementary Information). Rates of N application on UK agricultural grasslands vary, with a mean of 94.5 kg/ha^49^. The treatments at Park Grass (0, 48, 96 and 144 kg/ha) are, therefore, realistic and relevant. These treatment combinations have resulted in gradients of productivity, biodiversity and soil properties that can be analysed using regression models.

The 3 ha site is on a slightly acidic (pH 5.4–5.6), moderately well-drained, silty clay loam that was managed as permanent pasture for at least 100 years before the experiment began. The original vegetation (still represented on the ‘nil’ plots with no fertilisers added) was classified by Dodd et al.^50^ as dicotyledon-rich *Cynosurus cristatus-Centaurea nigra* grassland. The fertiliser treatments have acted as filters on the original community, with the species now found on each plot representing a naturally assembled community.

Park Grass is managed by making two hay cuts per year in June and October. We surveyed after the June cut because research indicates that competition for floral resources is weak in spring and strongest during high summer (i.e. July and August^14,51^), making this the key season in which increased floral resources would most benefit bees and other pollinators. Fieldwork began approximately one month after the June cut (20 June 2022, 22 Jun 2023) when the vegetation had recovered and resumed flowering. Four were control (zero fertilisers) and 28 treatment plots, half of which also received calcium carbonate, if needed, every three years to maintain a near-neutral soil pH (~6).

### Pollinator and Flowering Plant Censuses

We collected empirical data from 18 paired plots in 2022 (21 July to 2 September) and 32 paired plots in 2023 (9 July to 14 September). Transects were conducted by walking the perimeter of a plot and recording each interaction between an insect and flower within 2 m of the plot’s edge. Transects were conducted in the study plots between 10.00 and 16.00, during weather conditions suitable for pollinator activity (≥16°C and light wind). Park Grass study plots vary in area but are generally around 130 m^2^. In each plot we surveyed 40 m^2^ (20 x 2 m strip) 2-4 times per study day across 21 study days. Insects were identified to species using field-guides^52–54^ or assigned a morphospecies name. Care was taken not to record the same individual more than once per transect.

Flower abundance and species richness were quantified by counting the number of single flowers (e.g., *Lotus corniculatus*), stems (e.g., *Galium verum*) or inflorescences/capitulums (e.g. *Centaurea nigra*) as appropriate for all insect-visited species in bloom inside two 1m^2^ quadrats per transect, every 10 m. Flower species were identified using a field guide^55^. To standardise differences in flower abundance between species with differing flower sizes, the number of ‘flower units’ was calculated for each plant species per survey. A flower unit was defined as 1 cm^2^ with at least one open flower producing pollen or nectar, and was calculated for each species by multiplying flower abundance per survey by their respective petal area per flower/stem/inflorescence. Petal area per plant species was obtained from Balfour et al.^37^.

### Functional Traits

Pollinator functional traits (i) Order, (ii) wing length, and (iii) tongue length were characterised for each species. Wing length for each group was obtained from Ball and Morris^53^, Falk^54^, and Cook et al.^56^, and categorised as small (<5 mm), medium (5 – 10 mm) and large (>10 mm). Tongue length was obtained from Corbet^57^, Goulson et al.^58^, Gilbert^59^, and Balfour et al.^60^ and categorised as short (<8 mm), medium (8-9 mm), or long (>9 mm) following Goulson et al.^58^. For groups which were not identified to species, the average tongue and wing length was taken for that genus/family/order. All Dipterans were assumed to have short (<8 mm) tongues. Plant functional traits were characterised for each plant species: (i) flower shape, (ii) flower height, and (iii) petal colour. Flower shape and height were taken from Baude et al.^61^ and flower colour from Klotz et al.^62^.

Functional entities were generated for each unique combination of functional trait categories using the R package mFD^63^. The functional richness (total unique functional entities recorded) was calculated across the study period for each plot (Table S2, Supplementary Information).

### Soil properties

Soil nutrient data for each plot were provided by Rothamsted Research^64^: total N and C (% of total dry soil, dry combustion), plant available P (mg/kg of dry soil, Olsen-P method), exchangeable Ca, K, Mg, Na (mg/kg of dry soil, ammonium acetate method). Soil N and C were measured in March 2017, the others in March 2020. DEFRA Fertility Index was calculated for each study plot by taking the mean index score of P, K, and Mg^65^.

### Cost-Benefit Analysis

To determine the soil fertility that maximises both grassland productivity and flowering plant and pollinator species richness we combined the following data per study plot, for those receiving calcium carbonate: (i) mean Park Grass hay yield from 2022-2023 provided by Rothamsted Research^66^, (ii) the mean flowering plant and pollinator species richness recorded in this study, and (iii) the Park Grass soil nutrient data provided by Rothamsted Research^64^. To examine the trade-off between flower and pollinator species richness and grassland yield we normalised hay yields, flower species richness and pollinator species richness per plot, using the following formula: z_i_ = (x_i_ – min(x)) / (max(x) – min(x)).

### Legume Abundance

To determine the effect of soil nutrients on legume abundance, Fabaceae abundance (proportion biomass per plot) was obtained from Crawley et al.^24^ The data were collected during three study years between 1991-2000 from 40 plots at the Park Grass experiment across all four levels of lime application (Figure S3, Supplementary Information), the legumes species recorded were: *Lathyrus pratensis, Lotus corniculatus, Trifolium pratense, Trifolium repens*, and *Vicia sepium*.

### Statistical Analysis

All statistical analyses were conducted in R (version 4.2.2)^67^. Model assumptions were checked visually using the R package ‘DHARMa’^68^. For both the pollinator and flowering plant data we used Generalised Linear Mixed Models (GLMM) using the R package *glmmTMB*^69^. Prior to all statistical analysis, and the calculation of descriptive statistics, the two quadrats per transect were combined and all surveys were aggregated per study day.

Pollinator and flowering plant abundance and species richness (i.e. response variables) were all analysed separately. Date was included as a random factor. Models were simplified via stepwise removal of non-significant variables and likelihood ratio tests provided p values. Full models included mean DEFRA fertility index, N (%), and lime (applied or not) per study plot and their interactions as explanatory variables. Because the data were overdispersed, these GLMMs used negative-binomial (pollinator and plant abundance, pollinator species richness) and Poisson (plant species richness) error distributions. The final models for pollinator and flowering plant abundance and species richness included all explanatory variables. We used the ggpredict function from the ggeffects package^70^ to generate model predictions and confidence intervals for each of our GLMMs (Fig. 1). For the explanatory variable not shown (i.e. soil nitrogen or mean DEFRA Fertility Index) the ggpredict function assumes it to be the mean value across the dataset. For example in Fig. 1a the DEFRA Index is not plotted, therefore this variable is assumed to be the mean value across all plots (2.68).

To analyse the impact of soil nutrients on legume abundance, proportion biomass for all Fabaceae species were summed for each year and the mean calculated across the three study years. A GLMM was used to examine Fabaceae abundance (response variable), with a beta error distribution and a logit function. Mean DEFRA fertility index, N (%), and lime (not applied and three levels) per study plot were explanatory variables.

Separate GLMMs were used to examine honeybee abundance and non-honeybee abundance (response variables), with mean DEFRA fertility index, N (%), and lime (applied or not applied) per study plot as explanatory variables. The GLMMs for honeybee and non-honeybee abundance both used a Poisson and negative-binomial distribution respectively.

To examine whether pollinator and plant functional richness were related to plot treatments, GLMMs with functional richness as a response variable and N, DEFRA Fertility Index, and lime (applied or not) as explanatory variables were used. Both models had Gaussian error distributions. The number of surveys per plot was included as an offset in of both these models.

Beta regression, using the ‘betareg’ package^71^, was employed for the cost-benefit analysis. This tested the relationship between the response variables, mean hay yield, mean flowering plant species richness, and mean pollinator species richness per study plot, and the explanatory variable, soil fertility per plot (DEFRA Fertility Index). Linear models were used to test the relationship between flowering plant abundance and species richness per plot (explanatory variables), and pollinator abundance and species richness per plot (response variables).

## Supplementary information

Supplementary information

## Data Availability

The datasets analysed in this study are available from the corresponding author on request.

## Code availability

The underlying code for this study is not publicly available but are available from the corresponding author on request.
