# Supplementary material for: Trade-off between pollinator-wildflower diversity & grassland yields
Source: NPJ Biodivers. 2025 Jan 20;4:1. doi: 10.1038/s44185-024-00070-6 (PMC11756424; doi:10.1038/s44185-024-00070-6)
Supplement: Supplementary file 1 — Supplementary information [file 44185_2024_70_MOESM1_ESM.pdf]

Nicholas J. Balfour, Ciaran Harris, Jonathan Storkey and Francis L.W. Ratnieks

Supplementary Information

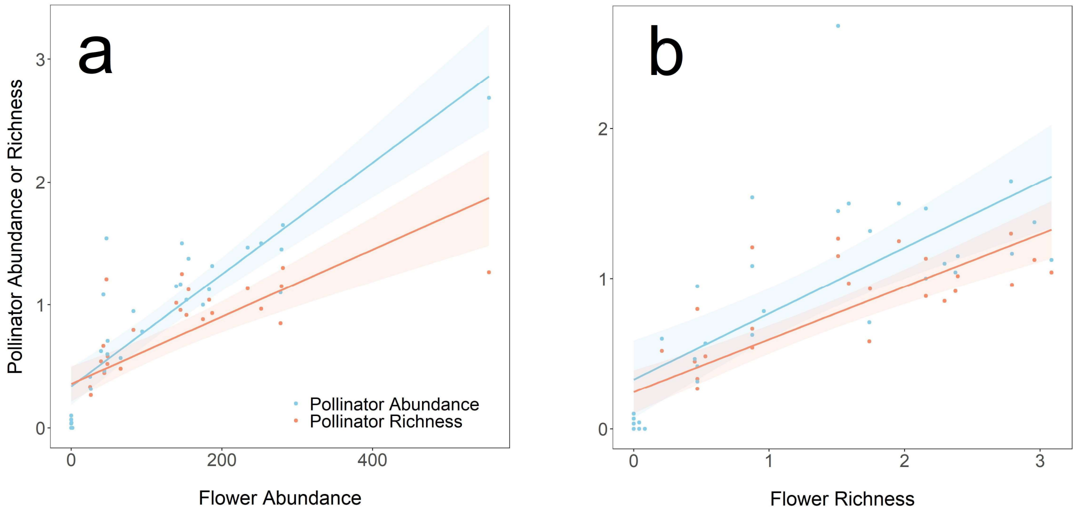

Figure S1 Variation in mean flower abundance (a) and species richness (b), and pollinator abundance (blue circles) and species richness (red triangles) per study plot. Shown are significant linear regressions (lines) and 95% confidence intervals (shaded areas). Flowering plant abundance per plot was significantly positively related to both pollinator abundance ($R^2 = 0.774$, t = 10.12, $P < 0.001$) and species richness ($R^2 = 0.59$, t = 6.51, $P < 0.001$) per plot (Figure S1a). Flowering plant species richness per plot was also significantly positively related to both and both pollinator abundance ($R^2 = 0.49$, t = 5.53, $P < 0.001$) and species richness ($R^2 = 0.67$, t = 7.93, $P < 0.001$) per plot (Figure S1b).

Nicholas J. Balfour, Ciaran Harris, Jonathan Storkey and Francis L.W. Ratnieks

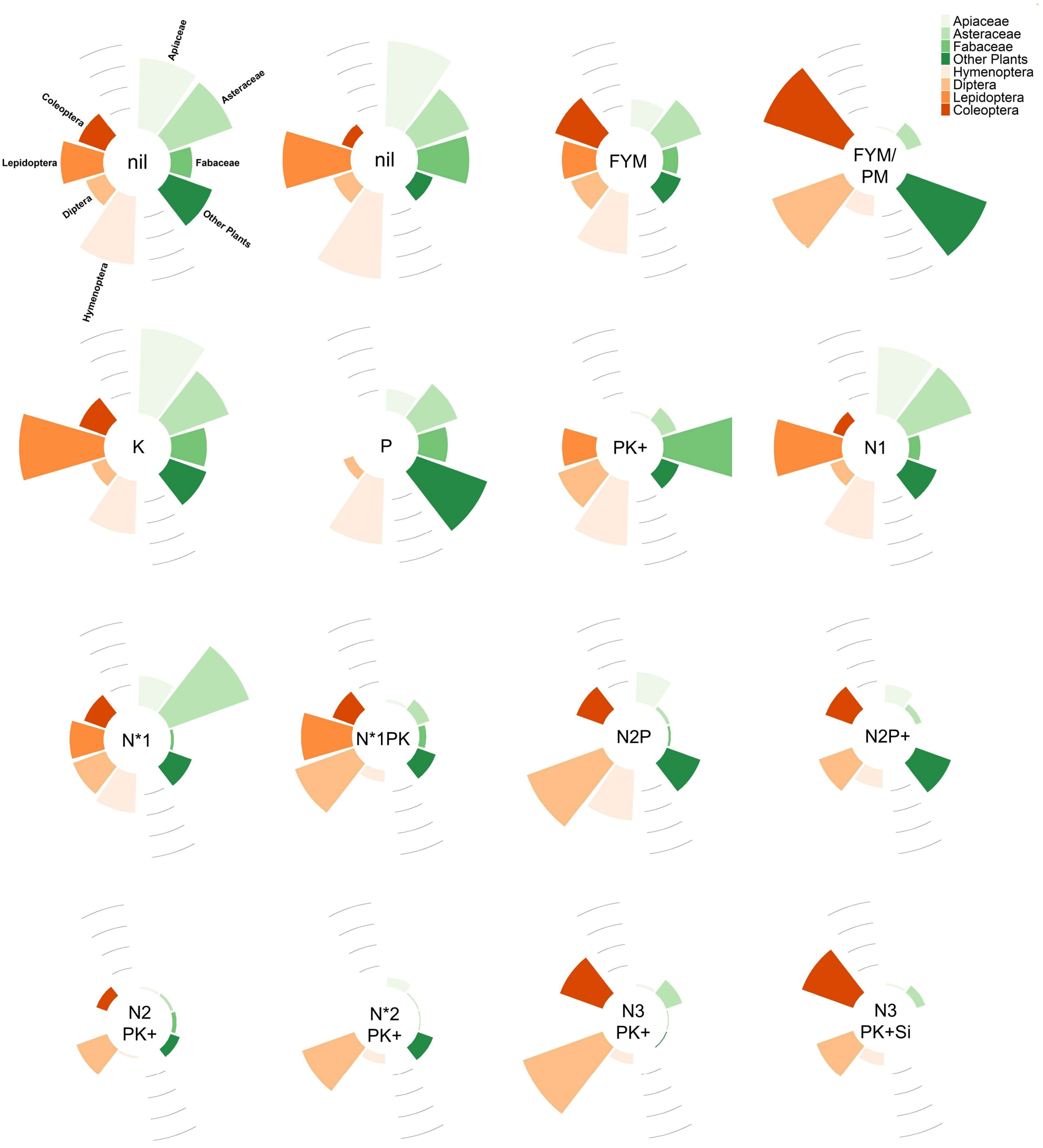

Figure S2 Radar diagrams showing the mean, per treatment plot: flower abundance of four groups (greens; Apiaceae, Asteraceae, Fabaceae, and other plants) and pollinator abundance of four orders (oranges; Hymenoptera, Diptera, Lepidoptera, and Coleoptera). Treatments are given in the middle of each radar diagram. Each category has been scaled against the maximum value recorded across the plots (i.e. x' = x/max(x)). Lines represent 0.25, 0.5, 0.75, and 1. See Figure A3 for details of treatments.

Nicholas J. Balfour, Ciaran Harris, Jonathan Storkey and Francis L.W. Ratnieks

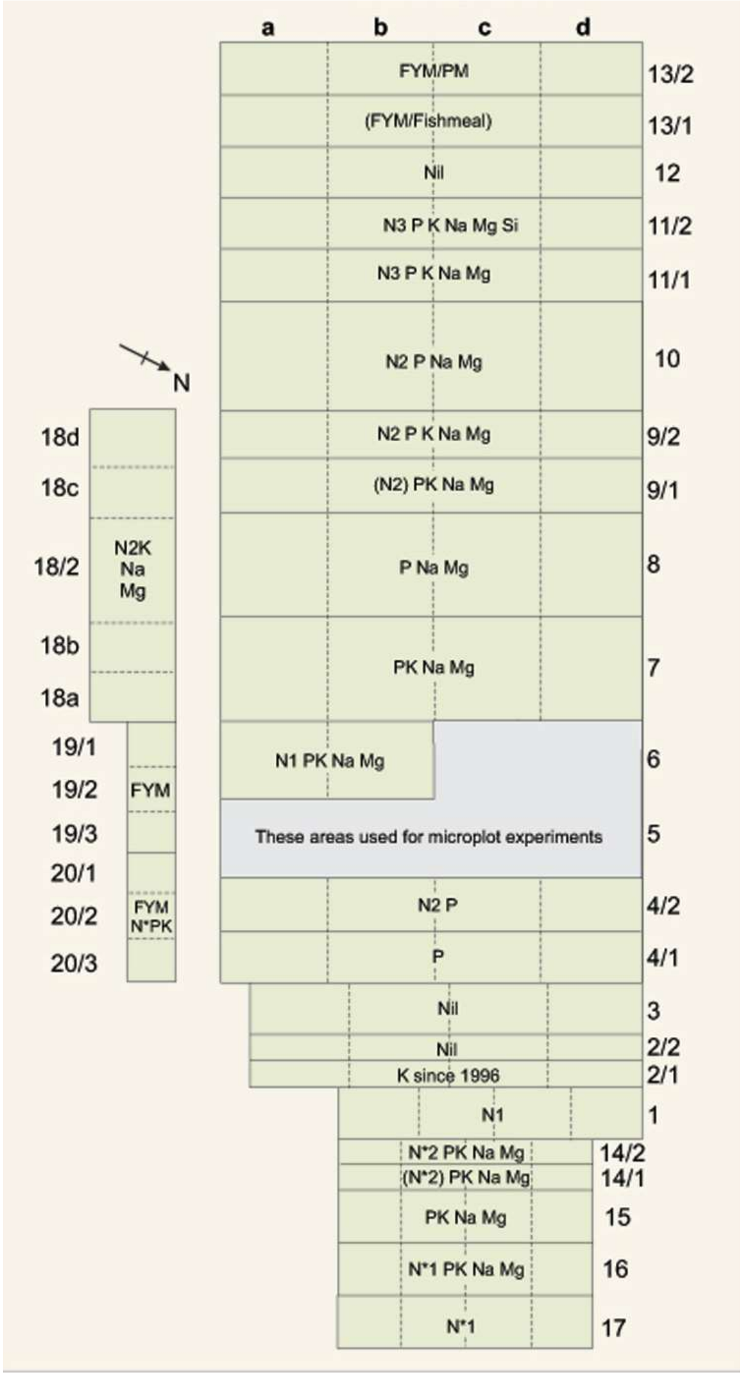

Figure S3 Layout of the Park Grass Experiment Rothamsted. Treatments: Nil plots: zero inputs, N*1, N*2: sodium nitrate supplying 48, 96 kg N/ha and 78, 157 kg Na/ha; N1, N2, N3: ammonium sulphate supplying 48, 96, 144 kg N/ha and 55, 110, 165 kg S/ha. Plus signs (+) indicate the addition of Na (sodium sulphate at 15 kg/ha Na and 10 kg/ha S) and Mg (magnesium sulphate at 10 kg/ha Mg and 13 kg/ha S). Si denotes water soluble sodium silicate supplying 135 kg/ha Si and 63 kg/ha Na. Organic fertiliser plots received 35 t/ha farmyard manure (FYM) and pelleted poultry manure (PM) every fourth year. Sub-plots a, b and c: differential amounts of chalk applied, if needed, every three years to maintain soil pH 7, 6 and 5, respectively. Sub-plot d receives no chalk. Image courtesy of Rothamsted Research.

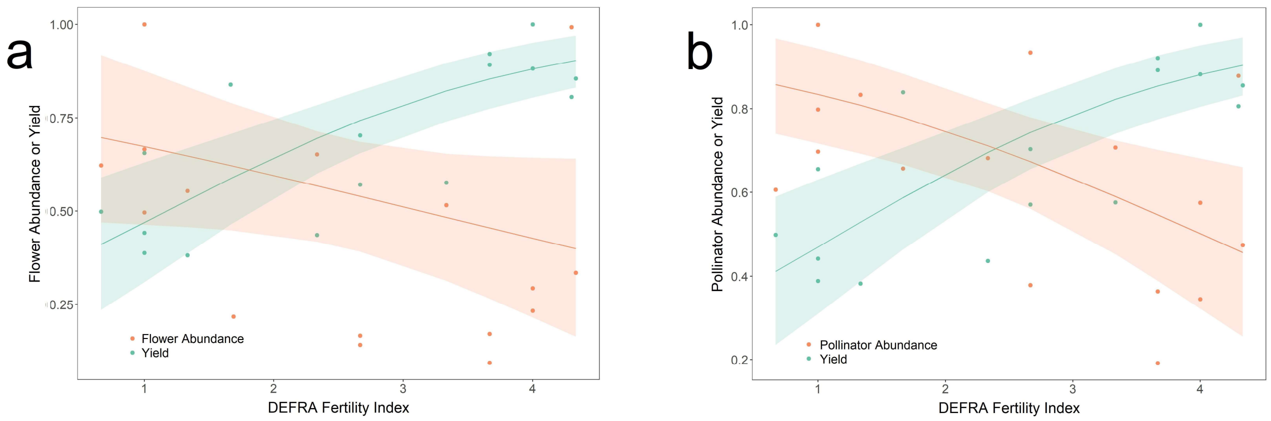

Figure S4 Relationship between flower (a) or pollinator (b) species abundance (orange circles), hay yield (green circles) and the DEFRA Fertility Index. Also shown are beta regressions (lines) and 95% confidence intervals (shaded areas). Yield (z =3.967, *P* <0.001) was found to be negatively correlated, flowering plant abundance (z = -1.420, *P* = 0.156) non-significantly related, and pollinator abundance (z = -2.646, *P* <0.001) significantly negatively, to the Defra Fertility Index to the Defra Fertility Index.

Supplementary Information: Trade-off between pollinator-wildflower diversity & grassland yields

Nicholas J. Balfour, Ciaran Harris, Jonathan Storkey and Francis L.W. Ratnieks

Table S1 Study Plots, Treatment (N*1, N*2: sodium nitrate supplying 48, 96 kg N and 78, 157 kg Na; N1, N2, N3: ammonium sulphate supplying 48, 96, 144 kg N and 55, 110, 165 kg S), Plot Number, Calcium Input (either none or lime applied, if needed, every three years to maintain soil pH 6), Mean Pollinator Abundance and Richness per 40 m^2 transect, Mean Flowering plant abundance and richness in two $1m^2$ quadrats. DEFRA Fertility Index score and hay yield (mean of 2022-23 data).

Treatment	Plot No	Calcium Input	Pollinator Abundance	Pollinator Richness	Flower Abundance	Flower Richness	DEFRA Index	Hay Yield
Nil	2/2b	Yes	1.375	1.125	155.667	2.958	1.33	3.85
Nil	2/2d	No	1.500	1.250	147.000	1.958	1.00	2.71
Nil	3b	Yes	1.650	1.300	281.158	2.784	1.00	4.45
Nil	3d	No	1.100	0.850	278.000	2.294	1.00	2.66
FYM/PM	13/2b	Yes	1.083	0.667	42.889	0.875	1.67	8.44
FYM/PM	13/2d	No	0.708	0.583	48.444	1.739	2.67	7.70
FYM	13/1b	Yes	1.317	0.933	187.211	1.745	1.00	6.59
FYM	13/1d	No	1.467	1.133	234.263	2.157	1.67	6.15
K	2/1b	Yes	1.125	1.042	183.222	3.083	2.33	4.39
K	2/1d	No	0.000	0.000	1.667	0.083	2.00	2.89
P	4/1b	Yes	1.167	0.958	145.111	2.792	3.33	5.79
P	4/1d	No	1.042	0.917	152.889	2.375	2.67	3.94
PKNaMg	7b	Yes	1.450	1.150	279.158	1.510	4.30	8.39
PKNaMg	7d	No	2.683	1.267	554.368	1.510	4.83	3.25
N1	1b	Yes	1.150	1.017	139.526	2.392	1.00	3.91
N1	1d	No	0.100	0.100	0.000	0.000	0.00	4.45
N*1	17b	Yes	1.000	0.883	175.053	2.157	0.67	5.02
N*1	17d	No	1.500	0.967	252.105	1.588	1.00	4.59
N*1PKNaMg	16b	Yes	0.783	0.783	94.316	0.961	4.33	8.61
N*1PKNaMg	16d	No	0.417	0.333	25.105	0.471	5.00	6.40
N2P	4/2b	Yes	1.542	1.208	46.778	0.875	2.67	5.74
N2P	4/2d	No	0.000	0.000	0.556	0.042	3.00	3.60
N2PNaMg	10b	Yes	0.625	0.542	39.778	0.875	2.67	7.07
N2PNaMg	10d	No	0.042	0.042	0.444	0.042	3.00	4.56
N2PKNaMg	9/2b	Yes	0.317	0.267	26.316	0.471	3.67	8.98
N2PKNaMg	9/2d	No	0.033	0.033	0.000	0.000	3.67	5.67
N*2PKNaMg	14/2b	Yes	0.569	0.483	65.579	0.529	4.00	8.88
N*2PKNaMg	14/2d	No	0.466	0.448	44.158	0.451	4.00	9.51
N3PKNaMg	11/1b	Yes	0.949	0.797	82.579	0.471	4.00	10.06
N3PKNaMg	11/1d	No	0.067	0.067	0.000	0.000	4.00	4.52
N3PKNaMgSi	11/2b	Yes	0.600	0.520	48.111	0.208	3.67	9.27
N3PKNaMgSi	11/2d	No	0.000	0.000	0.000	0.000	4.00	6.18

Nicholas J. Balfour, Ciaran Harris, Jonathan Storkey and Francis L.W. Ratnieks

Table S2 Output of generalised linear mixed models. Fixed factors were selected using a backwards stepwise selection process. Likelihood ratio tests provided p values for fixed factors. DFI = Defra Fertility Index.

`	Predictor variable	Coefficient	Standard error	χ^2	P
**Flower abundance**	Intercept	17.941	0.951		
	N	-42.311	2.879	66.881	<0.001 ***
	DFI	-0.536	0.084	26.282	<0.001 ***
	Lime	-9.608	1.486	111.75	<0.001 ***
	Lime: DFI	0.352	0.107	10.508	<0.001 ***
	Lime: N	33.359	4.296	58.632	<0.001 ***
**Flower species richness**	Intercept	6.575	0.467		
	N	-17.870	1.477	131.66	<0.001 ***
	DFI	-0.351	0.039	123.18	<0.001 ***
	Lime	-3.925	0.727	136.88	<0.001 ***
	Lime: DFI	0.111	0.049	5.050	0.025*
	Lime: N	14.423	2.212	40.947	<0.001 ***
**Flower functional richness**	Intercept	12.135	2.119		
	N	-31.088	5.985	13.388	<0.001***
	DFI	-0.200	0.287	0.481	0.49
	Lime	3.185	0.776	13.606	<0.001***
**Legume abundance**	Intercept	0.009	1.045		
	DFI	0.308	0.122	6.298	0.012*
	N	-11.377	3.115	7.478	0.006**
	Lime a	1.404	0.507	25.643	<0.001***
	Lime b	1.288	0.354		
	Lime c	2.225	0.500		
**Pollinator abundance**	Intercept	6.383	0.526		
	N	-17.990	1.642	79.912	<0.001 ***
	DFI	-0.164	0.033	13.452	<0.001 ***
	Lime	-4.088	0.879	65.097	<0.001 ***
	Lime: N	15.305	2.673	283.32	<0.001 ***
**Pollinator species richness**	Intercept	5.524	0.529		
	N	-15.607	1.589	64.600	<0.001 ***
	Fertility index	-0.214	0.045	16.373	<0.001 ***
	Lime	-3.797	0.826	74.483	<0.001 ***
	Lime: N	13.476	2.442	29.000	<0.001 ***
	Lime: DFI	0.115	0.058	3.925	0.048*
**Pollinator functional richness**	Intercept	9.133	1.987		
	N	-19.766	5.607	7.864	0.005**
	DFI	-0.566	0.269	4.132	0.03*
	Lime	2.448	0.727	9.239	0.002**
**Honey bee abundance**	Intercept	5.071	1.153		
	N	-19.975	3.111	57.274	<0.001***
	Fertility index	-0.733	00.245	45.09	<0.001***
	Lime	-9.223	1.428	1.294	0.003**
	DFI: N	-4.312	0.953	17.926	<0.001***
	Lime: N	29.371	4.200	55.977	<0.001***
	Lime: DFI	0.246	0.125	3.962	0.047*
**Non-honey bee abundance**	Intercept	3.352	0.385	7.008	
	N	-10.421	1.149	47.652	<0.001***
	DFI	-0.045	0.032	1.879	0.171
	Lime	1.024	0.118	79.039	<0.001***
	Lime: N	12.108	2.644	21.527	<0.001***
